# Functional diversity and metabolic engineering of plant-specialized metabolites

**DOI:** 10.1093/lifemeta/loac019

**Published:** 2022-08-25

**Authors:** Shaoqun Zhou, Yongshuo Ma, Yi Shang, Xiaoquan Qi, Sanwen Huang, Jiayang Li

**Affiliations:** Shenzhen Branch, Guangdong Laboratory of Lingnan Modern Agriculture, Genome Analysis Laboratory of the Ministry of Agriculture and Rural Affairs, Agricultural Genomics Institute at Shenzhen, Chinese Academy of Agricultural Sciences, Shenzhen, Guangdong 518120, China; Shenzhen Branch, Guangdong Laboratory of Lingnan Modern Agriculture, Genome Analysis Laboratory of the Ministry of Agriculture and Rural Affairs, Agricultural Genomics Institute at Shenzhen, Chinese Academy of Agricultural Sciences, Shenzhen, Guangdong 518120, China; Department of Chemical Engineering, Massachusetts Institute of Technology, Cambridge, MA 02142, USA; Yunnan Key Laboratory of Potato Biology, The CAAS-YNNU-YINMORE Joint Academy of Potato Sciences, Yunnan Normal University, Kunming, Yunan 650500, China; Key Laboratory of Plant Molecular Physiology, Institute of Botany, Chinese Academy of Sciences, Beijing 100093, China; Shenzhen Branch, Guangdong Laboratory of Lingnan Modern Agriculture, Genome Analysis Laboratory of the Ministry of Agriculture and Rural Affairs, Agricultural Genomics Institute at Shenzhen, Chinese Academy of Agricultural Sciences, Shenzhen, Guangdong 518120, China; State Key Laboratory of Plant Genomics, National Center for Plant Gene Research, Institute of Genetics and Developmental Biology, Innovation Academy for Seed Design, Chinese Academy of Sciences, Beijing 100101, China

**Keywords:** plant specialized metabolites, metabolic engineering

## Abstract

Plants are talented biochemists that produce a broad diversity of small molecules. These so-called specialized metabolites (SMs) play critical roles in the adaptive evolution of plants to defend against biotic and abiotic stresses, attract pollinators, and modulate soil microbiota for their own benefits. Many plant SMs have been used as nutrition and flavor compounds in our daily food, as well as drugs for treatment of human diseases. Current multi-omics tools have significantly accelerated the process of biosynthetic pathway elucidation in plants through correlation analyses, genetic mapping, and *de novo* biosynthetic gene cluster predictions. Understanding the biosynthesis of plant SMs has enabled reconstitution of naturally occurring specialized metabolic pathways in microbial hosts, providing a sustainable supply of these high-value molecules. In this review, we illustrate the general functions of several typical plant SMs in natural ecosystems and for human societies. We then provide an overview of current methods elucidating the biosynthetic pathways of plant SMs, and synthetic biology strategies that optimize the efficiency of heterologous biosynthetic pathways in microbial hosts. Moving forward, dissection of the functions and application of plant SMs by using current multidiscipline approaches would be greatly benefit to the scientific community and human societies.

## Introduction

Plants are superb biochemists that can produce a large diversity of small molecules by absorbing CO_2_, mineral elements, and water from the environment. These diverse metabolites are typically present in lineage-, developmental stage-, and tissue-specific manners, which are hence termed as specialized metabolites (SMs). The total number of structurally distinct SMs found across the plant kingdom has been estimated to be in the range of hundreds of thousands [1]. In contrast to the broadly conserved primary metabolites that are required for plant growth and development under ideal growth conditions, SMs play important roles in the interactions between plants and their environments. SMs add desirable flavors and important health benefits to human nutrition. In the last decades, molecular genetics and physiology studies have revealed biosynthesis of several important plant SMs. These knowledges have enabled synthetic biology approaches to reconstruct biosynthetic pathways of plant SMs in chassis organisms, producing the target compounds on an industrial scale. In this review, we briefly summarize the function of several typical plant SMs in natural ecosystems and for human consumption. We then delineate current methods in elucidating biosynthetic pathways of SMs in plants, and synthetic biology strategies to enhance target compound production in microbial chassis.

## SMs mediate plant–environment interactions

**Figure 1:**
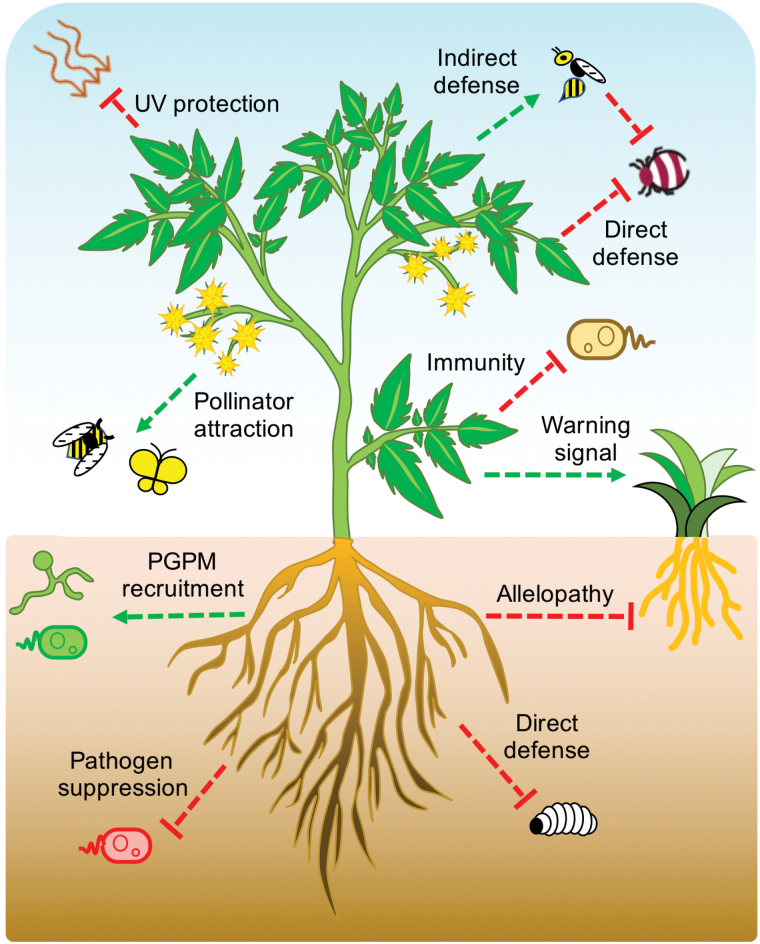
Plant SMs carry out diverse ecological functions. Attractions and repellence/toxicity are represented by green arrows and red T-shaped arrows, respectively. UV, ultraviolet; PGPM, plant growth-promoting microbes.

As sessile organisms, plants have adapted to a wide array of hostile environmental conditions. To cope with these challenges, plants produce diverse SMs to protect themselves from environmental stressors, attract animal pollinators for successful cross-fertilization, engineer local soil microbiota to support their own growth, and adapt to abiotic stresses. Examples illustrating these ecological functions of SMs are summarized in this section (Fig. 1).

### Protection of plants against insect herbivores and microbial pathogens

Plant SMs were initially appreciated for their protective functions against insect herbivores and pathogenic microbes. Classic examples of such compounds include glucosinolate [2], cyanogenic glucosides [3], nicotine [4], and benzoxazinoids [5]. Current mass spectrometry-based high-throughput analytical chemistry platforms and chemo-informatics tools have enabled exploration of more diverse defensive metabolites that present in low abundance in non-model organisms, as recently reviewed by Li and Gaquerel [6]. For example, a natural-history-guided omics approach has been taken to identify a novel compound, caffeoylputrescine-5-(*Z*)-3-hexenal, from a wild tobacco species, which was directly toxic to *Empoasca* leafhoppers when fed at the physiologically relevant concentration [7]. Interestingly, plants have also evolved exquisite detoxification mechanisms to avoid autotoxicity caused by the SMs they produce [8].

Other than being directly toxic against the intruding organisms, SMs could also protect plants by scavenging cytotoxic reactive oxygen species (ROS), fortifying the physical barriers of plants, altering the behavior of insect herbivores, and serving as environmental signal molecules. For example, many phenolic compounds could promptly remove excessive ROS in plant cells following the rapid ROS burst in response to environmental stimuli to avoid oxidative damage [9, 10]. Some phenolic compounds could also cross-link the cellulose fibers in plant cell walls to resist physical damage inflicted by insect attack and pathogen penetration [11]. Diverse classes of SMs including methyl salicylates [12], fatty acid derivatives [13], and terpenoids [14] have been demonstrated to either repel insect herbivores, or attract their natural enemies to protect the plants under attack. Some volatile compounds could also be perceived by neighboring plants as warning signals [15–21].

While plants are usually protected by the SMs that they produce, co-evolving pests have sometimes evolved measures to take advantage of plant SMs for their own benefits. For example, the hallmark SMs of maize (*Zea mays*), benzoxazinoids, are used by the specialized underground herbivore western corn rootworm (*Diabrotica virgifera*) as a reliable chemical signal to locate maize roots [22]. These specialized herbivores could not only tolerate the toxicity of benzoxazinoids, but also exploit the metal ion-chelating property of these compounds to supplement iron acquisition in their diets [23], and sequester the toxic compounds to protect themselves from natural enemies [24]. The delivery of plant SMs through complex food chains further extends the ecological significance of these natural products. Indeed, a recent experimental ecology study has demonstrated that allelic variation in a plant-specialized metabolic gene could enhance the persistence of the local food web [25].

### Attraction of animal pollinators

The evolutionary success of flowering plants has been associated with insect-mediated cross-pollination. While the fundamental rewards provided to insect pollinators are primary metabolites such as carbohydrates (i.e. nectar) and proteins (i.e. pollen), SMs play critical roles in pollinator attraction by adding colors and fragrances to the floral display [26, 27]. From early field ecology observations to recent molecular genetics dissections, studies in plant–pollinator interactions have transcended multiple biological scales. A classic example was illustrated by a series of studies on *Mimulus* species. Bradshaw [28] identified the *YELLOW UPPER* locus, which influenced the petal color by regulating tissue-specific carotenoid metabolism, and this variation in floral coloration was associated with either bees or hummingbirds as the predominant pollinators in their natural habitats. The authors then hybridized these species to generate an experimental near iso-genic population with the different *YELLOW UPPER* alleles introgressed into the reciprocal genetic background, and demonstrated that the native pollinator visitation rate was negatively influenced by this introgression [29, 30]. More examples demonstrating the function of plant SMs in plant–pollinator interactions have been elegantly synthesized in a recent review by Fattorini and Glover [31]. Interestingly, some pollinator-attracting plant SMs could also exert dual functions as a defensive metabolite against florivorous insects [32].

### Modulation of rhizospheric microbiota

As early as the first century ad, it was observed that some plants, such as black walnut (*Julgans nigra*), were capable of inhibiting the growth of neighboring plants, a phenomenon known as allelopathy. It has since been shown that allelopathic interactions between plants are mediated by toxic SMs exuded into the rhizosphere [33]. In the last decades, researchers have demonstrated that root exuded SMs are not limited to toxic compounds, but could rather function as critical chemical messengers that facilitate interactions between plants and soil microbes. Textbook examples on this topic include fabaceous flavones, which induce expression of nodulation genes in *Rhizobium* bacteria [34], and strigolactones, which stimulate branching of germinating arbuscular mycorrhizae fungi [35]. More recently, technological advances in environmental DNA sequencing and microbe culturing have revealed the robust dynamics of plant–microbiota interactions in the rhizosphere. To date, the rhizospheric microbiota-engineering function has been demonstrated for a long list of exuded SMs including benzoxazinoids [23, 36], phenolics [37, 38], and terpenoids [39, 40]. One of the less studied topics in this area is how the SMs in root exudate are exported on the cellular level. In the model species *Arabidopsis thaliana*, exudation of coumarins was facilitated by an ATP-binding cassette-containing transporter [41]. Recent evidences from our group suggest that a Multidrug and Toxic Compound Extrusion transporter expressed in melon (*Cucumis melo*) roots is responsible for the exudation of a triterpene compound, cucurbitacin B, which can suppress soil-borne fungal pathogens by enriching specific taxa of beneficial bacteria [42].

### Adaptation toward abiotic stresses

In addition to protecting plants against biotic stressors, SMs also participate in the plant adaptive processes toward various abiotic stresses. For example, enhanced accumulation of flavonoids in Tibetan barley (*Hordeum vulgare,* qinke) was proposed to be an adaptive mechanism to protect against exacerbated ultraviolet-B irradiation of the Tibetan plateau [43]. Concentrations of various SMs were significantly altered in maize plants grown under low-phosphorus conditions [44]. Most recently, genome-wide association studies followed by candidate validation with genetic mutant analyses have revealed the functionality of a number of maize SMs in drought and salt resistance [45, 46].

## SMs used as flavor, nutrition, and medicine

**Figure 2:**
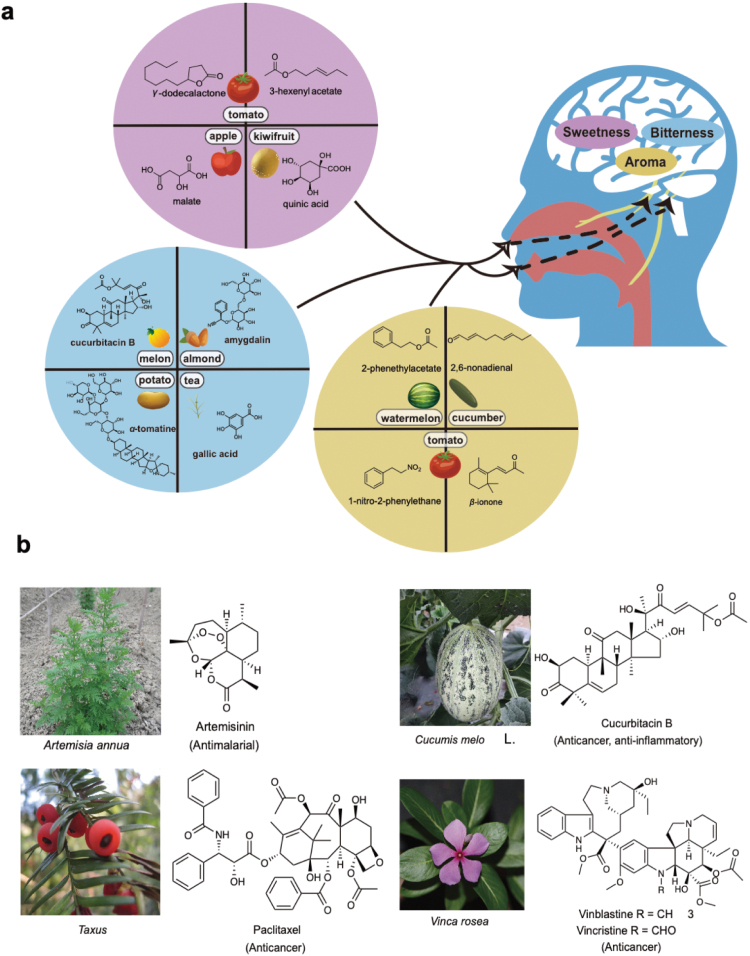
Flavoring and medicinal values of plant SMs for humans. Representative plant SMs and their natural botanical sources are used as flavors (a) and medicines (b).

Plants are the primary producers of our ecosphere. While the primary metabolites in plants provide essential nutrients to humans, plant SMs are valued for their flavoring, nutraceutical, and medicinal properties. Current metabolomics methods have enabled researchers to pinpoint and quantify the keystone plant metabolites that are associated with consumer preference or specific therapeutic functions. Some of these examples are delineated in this section (Fig. 2).

### Flavors

Sweetness is perhaps one of the most desirable traits for freshly consumed fruits and vegetables (Fig. 2a). Recent studies in tomato (*Solanum lycopersicum*) and blueberry (*Vaccinium corymbosum*) have demonstrated that the sense of sweetness is not only associated with sugar content, but also promoted by various volatile compounds [47, 48]. In contrast, organic acid contents have been reported to be negatively correlated with perceived levels of sweetness in tomato [48], apple (*Malus* spp.) [49–51], and kiwifruit (*Actinidia arguta*) [52, 53]. The complex biochemical composition associated with the sense of sweetness provides the opportunity to improve this important quality trait by targeting the SMs of these crops rather than directly manipulating their sugar metabolism, so as to avoid any potential yield penalty. Furthermore, some plant SMs, such as stevia glycosides and mogrosides, are inherently sweet, and are widely exploited as natural low-caloric sweeteners. Besides sweetness, different fruits and vegetables often have their characteristic tastes and aroma, which are also endowed by their SMs [54]. Distinctive aroma of cinnamon (*Cinnamomum verum*) [55], melon [56], cucumber (*Cucumis sativa*) [57, 58], pumpkin (*Cucurbita* spp.) [59], and kiwifruit [60, 61] are determined by their characteristic volatile phenolics and fatty acid derivatives. A variety of volatile terpenoids and uncommon nitrogen-containing volatiles have also been shown to contribute to the aroma of tomato [62–65].

Since SMs mainly function as defensive compounds for plants in nature, most SMs are toxic and/or have unpleasant taste to humans [66, 67] (Fig. 2a). The presence of these SMs in the edible parts of crop plants has often been negatively selected during domestication. In cucurbits, a mutation in a fruit-specific transcription factor abolished the biosynthesis of bitter-tasting triterpenoids in fruits [68]. Similar changes in tissue-specific transcription regulation have recently been demonstrated to lower the level of toxic cyanogenic glycosides in domesticated almond kernels [69]. In tomato fruits, a tissue- and developmental stage-specific nitrate/peptide family transporter could export the bitter α-tomatine from vacuole to cytosol during fruit ripening for prompt degradation [70]. On the other hand, some SMs that are nontoxic but have pungent tastes to human are preserved during crop domestication. For example, assorted phenolic acids contribute to bitterness and astringency in tea leaves (*Camellia sinensis*), which contribute to the complex flavor of tea [71, 72]. In olive fruits (*Olea* spp.), the content of bitter-tasting oleuropein needs to be properly controlled during olive breeding for a balanced taste in the fruits [73, 74].

While SMs can directly affect the taste and aroma of freshly consumed fruits and vegetables, food processing for most staple crops has a significant impact on the biochemical composition, and hence the flavor of food. Maillard reaction is one of the most common chemical reactions that occurs during food processing, which produces ﬂavorful volatiles [75]. For example, furans contribute the caramel-like odor of heated carbohydrates, and pyrazines and methional provide the characteristic aroma of baked potatoes [76].

### Nutraceuticals and medicine

Besides serving as flavor molecules, some plant SMs, such as vitamins, are essential components of human nutrition. Vitamin C (l-ascorbic acid) is the most classic example of essential nutrient that is exclusively supplied by fresh plants since humans and other primates have lost their own capacity to biosynthesize this vital molecule [77]. Similarly, plants are an important source of diverse B vitamins including B1 (thiamin from grains), B5 (pantothenic acid from sunflower seeds and potatoes), and B9 (folate from spinach) [78–80]. Recent technological advances in genetic engineering and genome editing have inspired a series of bio-fortification efforts to enhance the nutritional quality of staple crops, for more accessible nutrition in under-developed regions. For example, five carotenoid biosynthetic genes have been specifically expressed in maize kernels to promote accumulation of a variety of carotenoids, which can be converted to vitamin A in animals after ingestion [81]. Chickens raised on these bio-fortified feed produced high-carotenoid poultry meat and were more resistant against a protozoan parasite [82]. Most recently, a specific isoform of 7-dehydrocholesterol reductase in tomato was knocked-out to allow accumulation of 7-dehydrocholesterol, which would be simultaneously converted into vitamin D upon ultraviolet irradiation [83].

Plant SMs have been the active ingredients of many traditional medicines for millennia. In the last 2 centuries, detailed analytical chemistry studies have revealed the principal active compounds associated with various medicinal plants (Fig. 2b). For example, the dried aerial parts of the herb *Artemisia annua* have been used in China for centuries to treat fever and malaria [84]. The main active compound, artemisinin, is highly effective against malaria parasites *Plasmodium falciparum*, without being toxic to humans or animals at the same concentration [85]. Cucurbitacins, a class of triterpenoids widely distributed in cucurbit plants, have been exploited for their anti-inflammatory, hepatoprotective, and antiproliferative effects [86]. Paclitaxel, a diterpene from *Taxus* species, is a well-known chemotherapy agent against various cancers [87]. Other classic chemotherapeutic compounds, vinblastine and vincristine, are naturally sourced from *Vinca rosea* for treatment of Hodgkin’s lymphoma and childhood lymphoblastic leukemia [88]. More of the current progress and future perspectives of the use of plant SMs as a source of medicine have been recently reviewed by Jacobowitz and Weng [89].

## Elucidating biosynthetic pathways of SMs in plants

**Figure 3:**
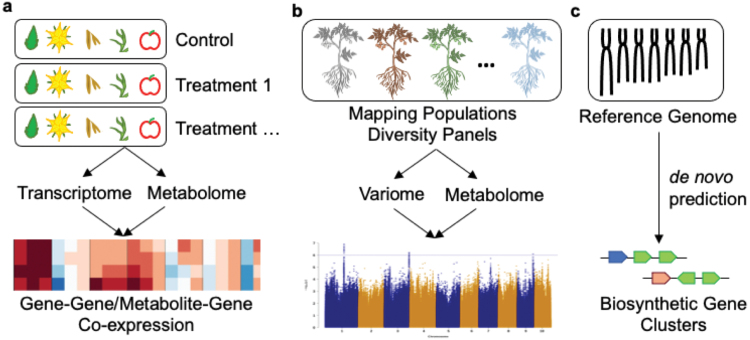
Current methods for deciphering biosynthetic pathways of plant SMs. Schematic representation of (a) correlation analyses based on multi-tissue and multi-treatment transcriptome and metabolome data; (b) forward genetics with *ad hoc* mapping populations and/or genetic diversity panels; (c) *de novo* prediction of operon-like BFCs in reference-grade genomes.

Since plant SMs are appreciated for their flavoring and therapeutic functions for human, understanding the biosynthesis of SMs has always been one of the top priorities in plant biology research. Early efforts to clarify plant-specialized metabolism relied on traditional biochemistry techniques such as stable isotope feeding and protein chromatography coupled with *in vitro* enzymatic assays. In the last decade, rapid development in multi-omics tools have enabled a tremendous leap in specialized metabolic pathway elucidation in plants. This section summarizes studies exemplifying these current methods (Fig. 3).

### Metabolite-gene expression correlation

Plant SMs are well-known for their tissue-specific distribution and inducible accumulation. This spatiotemporal heterogeneity allows researchers to identify potential biosynthetic genes by looking for statistical correlation between experimentally determined gene expression and target metabolite abundance (Fig. 3a). Since the 2010s, this simple yet effective approach has been successfully applied to identify key biosynthetic enzymes of a number of renowned plant SMs such as podophyllotoxin [90], vinblastine and vincristine [88], and colchicine [91, 92]. This method also contributed to the recent characterization of root-specific triterpenoid biosynthetic pathways in *Arabidopsis* [39], and proto-limonoid biosynthesis in rutaceaeous plants [93]. One of the key advantages of this approach is that it only requires a few samples for transcriptomics and targeted metabolomics analyses to identify potential candidate genes. For example, it took 12 RNAseq samples to identify the candidate biosynthetic genes of podophyllotoxin through a co-expression analysis [90]. Also, transcriptome data can be *de novo* assembled for quantitative analyses, which bypasses the requirement to sequence and assemble large plant genomes. These advantages have significantly enhanced the practical viability of correlation analyses in genetic dissection of plant-specialized metabolism, especially in non-model medicinal plant species that lack diverse germplasms and reliable reference genomes. However, it should be noted that some SMs are transported away from their main tissues of biosynthesis and stored elsewhere (e.g. nicotine, cardenolide), which would hence nullify the presumed cross-tissue correlation of the abundance of the SM with the expression of its biosynthetic genes.

### Forward genetics

In addition to studies of rare and medicinal SMs in non-model plant species, examination of SMs in staple and economic crops is another fast-developing area in plant research. Here, thanks to the diverse germplasm and genotypic information accumulated from generations of crop breeders, the forward genetics approach is preferred to identify the biosynthetic and/or regulatory genes associated with the target SMs (Fig. 3b). In maize, e.g. classic bi-parental mapping populations are now routinely adopted to locate the genetic loci associated with SMs [94–98]. Though genetic mapping with bi-parental populations alone rarely gets down to single-gene resolution, addition of a clear biochemical hypothesis and gene expression data are usually sufficient to narrow down the list of candidate genes to a range that can be experimentally validated.

Compared to bi-parental populations, the more diverse genome-wide association panels offer much more frequent recombination, and hence much higher mapping resolution. Indeed, studies in rice (*Oryza sativa*) [99], maize [100, 101], and tomato [48, 102] have demonstrated that single-gene level mapping can be achieved with sufficient molecular marker coverage. On the contrary to what was once commonly believed, high-resolution association genetics revealed that the genetic architecture of plant SMs is not necessarily simpler than that of composite traits such as grain yield [101]. This is probably reflective of the complex *cis* and *trans* regulation of specialized metabolism in plants. Nevertheless, current genetic dissection of SM biosynthetic pathways in crop species have demonstrated the potential to enhance crop quality and to protect crop plants against environmental stresses through genetic engineering [102, 103].

### Genome-mining based on operon-like gene clusters

Since the first report of a cluster of five genes (Bx1 through Bx5) that are required for synthesis of antimicrobial compound, 2,4-dihydroxy-1,4-benzoaxin-3-one (DIBOA), at a narrow region on a maize chromosome [104], another metabolic gene cluster for biosynthesis of antimicrobial compounds, avenacins, was defined by genetic and bacterial artificial chromosme analysis in oats [105, 106]. Since then, similar biosynthetic gene clusters (BGCs) have been reported across diverse plant species for the production of many plant SMs [107] (Fig. 3c; Table 1). A potential evolutionary advantage of these BGCs in plant genomes is that the physical proximity between these enzyme-encoding genes could facilitate shared epigenomic modifications and hence coordinated expression regulation [67, 68, 131–133]. For example, tissue-specific *trans* regulators can directly bind to the conserved E-box elements in the promoters of nine clustered cucurbitacin biosynthetic genes, and coordinately activate their transcriptions to produce the target compound [67, 68]. Interestingly, the later studies have demonstrated that BGCs can include not only enzyme-encoding genes, but also genes that encode specific transporters of the SMs produced [134, 135].

**Table 1:** Examples of characterized plant BGCs.

Compound(s)/pathway	Class	Plant species	Role in plant	References
Avenacins	Triterpenes	*Avena* sp.	Antifungal	[8, 105]
Arabidiol/arabidin	Triterpenes	*Arabidopsis thaliana*	Anti-oomycete, microbiome modulation	[39, 108]
Thalianol/thalianin	Triterpenes	*A. thaliana* *Arabidopsis lyrata*	Microbiome modulation	[39, 108, 109]
Marneral	Triterpenes	*A. thaliana*	Unknown	[108]
Tirucallol	Triterpenes	*Capsella rubella*	Unknown	[109]
Euphol	Triterpenes	*Brassica rapa*	Unknown	[109]
Cucurbitacins	Triterpenes	*Cucumis sativus* *Cucumis melo* *Citrullus lanatus*	Antibacterial, antifungal, insecticidal, anti-herbivore	[68, 110]
Yossosides	Triterpenes	*Spinacia oleracea*	Unknown	[111]
20-hydroxy-betulinic acid	Triterpenes	*Lotus japonicus*	Unknown	[112]
Momilactones	Diterpenes	*Oryza* sp. *Echinochloa crusgalli* *Calohypnum* *plumiforme*	Antibacterial, antifungal, allelopathic	[113, 114]
Phytocassanes/oryzalides	Diterpenes	*Oryza sativa*	Antibacterial, antifungal	[115]
Casbene diterpenoids	Diterpenes	*Ricinus communis* *Euphorbia peplus* *Jatropha curcas*	Antifungal, antibacterial	[116]
5,10-diketo-casbene	Diterpenes	*O. sativa*	Antifungal, antibacterial	[117]
Various monoterpenes and diterpenes	Diterpenes/monoterpenes	*Solanum* sp.	Antibacterial, antifungal	[118]
Lycosantanolol	Diterpenes	*Solanum lycopersicum*	Unknown	[119]
5-*epi*-jinkoheremol/ debneyol	Sesquiterpenes	*Catharanthus roseus*	Fungicidal activity	[120]
α-tomatine	Steroidal glycoalkaloids	*S. lycopersicum*	Antibacterial, antifungal, insecticidal	[121]
α-solanine α-chaconine	Steroidal glycoalkaloids	*Solanum tuberosum*	Antibacterial, antifungal, insecticidal	[121]
Noscapine	Benzylisoquinoline alkaloids	*Papaver somniferum*	Unknown	[122]
Thebaine	Benzylisoquinoline alkaloids	*P. somniferum*	Unknown	[123]
Hydroxycinnamoyl-tyramine conjugates	Phenolamides	*O. sativa*	Antibacterial, antifungal	[124]
Feruloylputrescine	Phenylpropanoids	*O. sativa*	Immunity, cell death	[125]
Dhurrin	Cyanogenic glucosides	*Sorghum bicolor*	Insecticidal, anti-herbivore	[3]
Linamarin Lotaustralin	Cyanogenic glucosides	*L. japonicus Manihot esculenta*	Insecticidal, anti-herbivore	[126]
α-/β-/γ-Hydroxynitrile glucosides	Hydroxynitrile glucosides	*Hordeum vulgare*	Unknown	[127]
Falcarindiol	Fatty acids	*S. lycopersicum*	Antifungal, antibacterial	[128]
β-diketones	Polyketides	*H. vulgare* *Triticum turgidum*	Forming physical barrier on leaf surface	[129]
DIBOA/DIMBOA	Benzoxazinoids	*Zea mays*	Antibacterial, antifungal, insecticidal, allelopathic	[104]
Various acylsugars	Acylsugars	*Solanum* sp.	Antifungal, insecticidal, anti-herbivore	[130]

As the idea of plant BGCs receives accumulating experimental support, bioinformatic tools that predict BGCs solely based on genomic information have also been developed to annotate previously unknown SM biosynthetic genes [136]. A recent study on wheat (*Triticum aestivum*) and a related model grass species exemplified this reverse chemical genetics approach, where the authors started by annotating potential BGCs in recently published genomes and ended up identifying previously unknown SMs in these species by combining pathogen-inducible gene expression data and functional validation experiments [137].

It is noteworthy that the functionally related but structurally non-homologous plant BGCs bear close resemblance to prokaryotic operons, since members of both BGCs and operons are tightly linked in the genomes and tend to be co-expressed [138]. That being said, BGCs and operons most likely arise independently during evolution, given the distant phylogenetic separation between plants and bacteria. While prokaryotic operons are well-known to horizontally transfer between co-habituating microbes, plant BGCs tend to arise independently in each phylogenetic lineage which then go on to differential evolutionary trajectories [67, 139–141]. However, recent studies of the classic benzoxazinoid BGCs in maize and wheat suggested that this cluster may have been horizontally transferred between their ancestors, followed by independent evolution of a functionally homologous downstream modification enzyme [142, 143]. It will take further studies to determine whether this proposed horizontal BGC transfer is an isolated case or a harbinger of a more common pattern in plant evolution.

## Synthetic biology for sustainable supply of plant SMs

**Figure 4:**
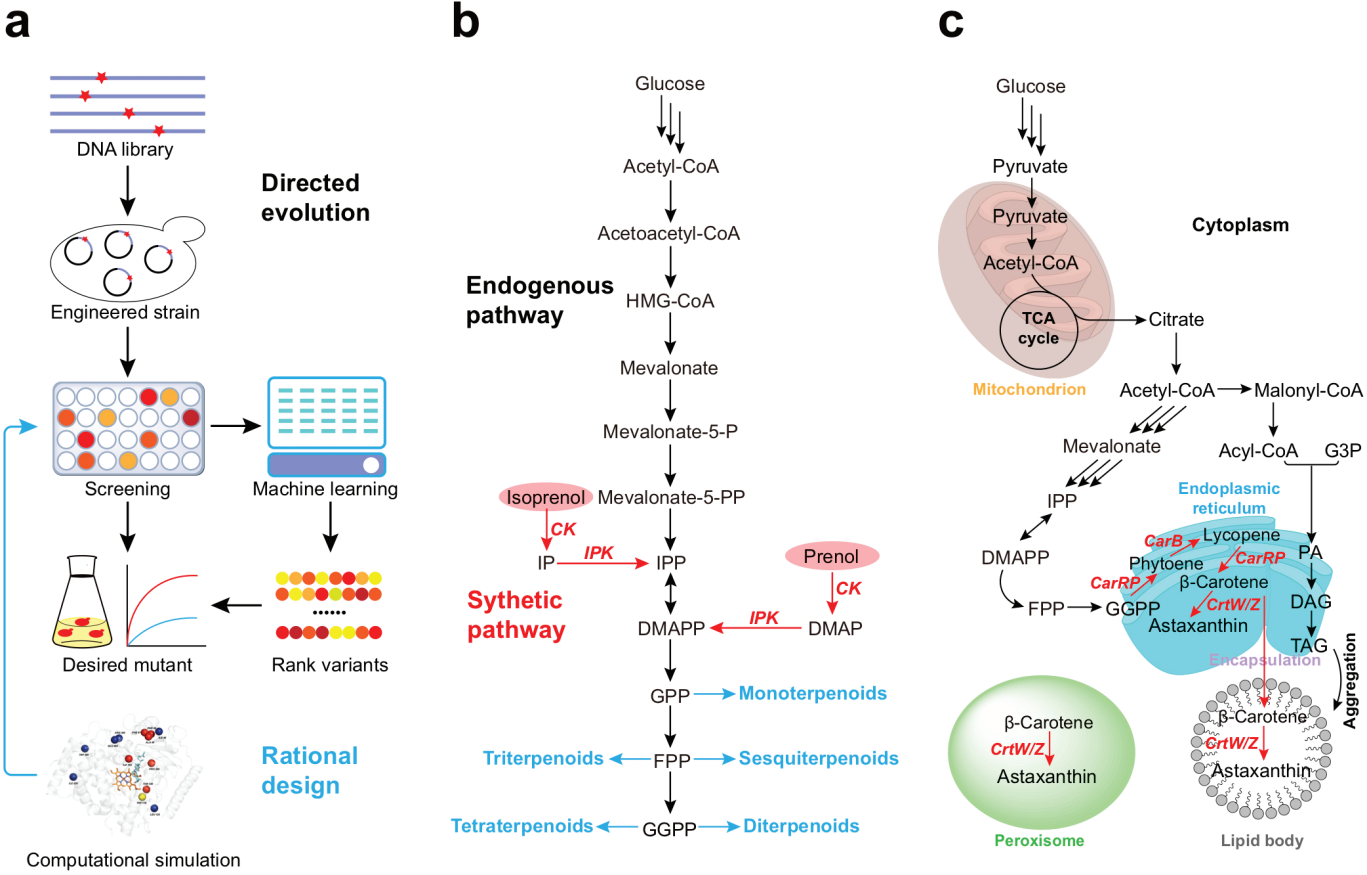
Synthetic biology strategies for enhanced plant SM production in microbial chasses. Schematic representation of (a) enzyme engineering through directed evolution or rational design; (b) introduction of synthetic orthogonal pathways; (c) compartmentalization of synthetic pathways.

Though plant SMs are widely utilized by human beings, the low abundance of valuable SMs *in planta* and the low efficiency in the extraction process limit their supply from native sources. The expanding inventory of functionally characterized plant enzymes, coupled with advancing synthetic biology tools, provides opportunities to sustainably produce plant SMs in engineered chassis microbes such as *Escherichia coli* and *Saccharomyces cerevisiae* [109, 144–148]. However, heterologous plant SM biosynthetic pathways in microbes often perturb the innate metabolic balance of the host, resulting in lowered target productivity or even host fatality [149]. Therefore, fine-tuning of the heterologous pathways is essential to unlock the full potential of microbial cell factories for plant SM production. Here we summarize several representative synthetic biology strategies in optimizing the native metabolic pathways of chassis microbes for enhanced production of heterologous compounds (Fig. 4).

### Enzyme engineering (design and generation of highly efficient enzymes)

On the most basic molecular level, the efficiency of a metabolic pathway depends on the catalyzing efficiency of the enzymes. Assembly of naturally occurring plant enzymes into heterologous biosynthetic pathways in chassis organisms often suffers from lowered catalytic activity, narrowed substrate specificity, poor protein structure stability, and unexpected allosteric inhibition [150]. Traditionally, these problems had been addressed by increasing the titer of the heterologous enzymes with optimized gene expression levels or gene copy numbers, as well as utilization of superior alternative enzymes from other natural sources [151]. More recently, protein engineering methods such as directed evolution and site-directed mutagenesis have emerged as more efficient approaches to enhance the performance of rate-limiting enzymes [152] (Fig. 4a).

Directed evolution is one of the earliest protein engineering approaches as it does not require detailed knowledge of protein structure. By reiteratively selecting for the most efficient structural variant of the enzyme under study, this process enhances overall enzyme performance through evolution by selection. This approach has been adopted to enhance astaxanthin and various isoprenoids production in yeast and *E. coli* [153–156]. However, it should be noted that successful cases of enzyme engineering through directed evolution are almost exclusively reported for carotenoid-related pathways, as the distinctive pigmentation of the end product provides an easy proxy for high-throughput selection [157]. More recently, researchers have attempted to circumvent this limitation by incorporating machine-learning algorithms into directed evolution schemes to reduce the sequence space that had to be experimentally tested [158, 159].

The rapid development of crystallography-based structural biology has enabled structure-guided protein engineering by computer-assist molecular simulation. Through this so-called site-directed mutagenesis approach, key amino acid residues that affect enzyme activities can be identified by resolving the crystal structure of the target protein and simulating possible molecular interactions. The protein structure can then be optimized by designing proper amino acid substitution schemes [150]. For example, modification of a glycosyltransferase through structure-based rational design achieved a 1800-fold increase in catalytic efficiency for the production of ginsenoside Rh2, a potential anticancer compound [160]. This approach was also applied to improve substrate selectivity and protein stability of enzymes [161]. One of the main bottlenecks for broader application of site-directed mutagenesis of enzymes is the difficulty to obtain precise protein structures experimentally. Recent progress in computational protein structure prediction algorithms (e.g. AlphaFold and RoseTTA-Fold) has significantly promoted the prediction accuracy, reaching the same level of accuracy as the experimentally resolved protein structures in many cases [162, 163]. This combination of prediction accuracy and practical flexibility could enable site-directed improvement of more biosynthetic enzymes of plant SMs.

### Synthetic orthogonal pathways (modulation of microbial chassis)

While targeted protein engineering could enhance the metabolic flux within the engineered SM pathways, the metabolic bottlenecks in microbial chassis could also be positioned further upstream, limiting the supply of chemical precursors. In these cases, synthetic orthogonal pathways are built to provide an additional route of precursor supply without perturbing the native pathway to avoid undesirable side-effects [164] (Fig. 4b). This strategy has been widely adopted in the case of heterologous terpenoid production, where the canonical terpene precursors are tightly coupled to sterol biosynthesis essential for the survival chassis organisms. Although heterologous introduction of the entire mevalonate pathway into *E. coli* to replace the native methylerythritol 4-phosphate pathway is now commonly adopted [165–167], both pathways are strongly coupled with the central carbon metabolism and are tightly regulated, thus limiting the availability of the terpenoid precursors, isopentenyl pyrophosphate (IPP) and dimethylallyl pyrophosphate (DMAPP). Recently, a synthetic orthogonal pathway was developed to produce IPP and DMAPP from isoprenol or prenol through two steps of phosphorylation [168]. This synthetic Isopentenol Utilization Pathway (IUP) is decoupled from the central carbon metabolism of *E. coli,* and hence can sustain a high-carbon flux toward terpene precursors without disturbing the normal metabolism of the chassis microbe. In addition, IUP only uses ATP as the sole co-factor, further reducing the potential impact of this synthetic pathway on the host metabolism through co-factor competition. A similar approach of orthogonal IPP and DMAPP production from glycerol has also been reported, which eliminated the need to feed isopentenols in the culturing medium [169]. These orthogonal pathways have managed to enhance heterologous isoprenoid production in various microbial systems [168–171]. In another example, introduction of a heterologous geranyl diphosphate (GPP) synthase using neryl diphosphate as the predominant substrate has enhanced GPP production compared to the native yeast prenyltransferase, which in turn promoted the heterologous monoterpene accumulation [164].

### Co-compartmentalization of biosynthetic pathway

In plants, specialized metabolism occurs in specific subcellular locations to promote metabolic efficiency and avoid autotoxicity by bioactive intermediates [89]. Inappropriate localization of a heterologous biosynthetic pathway in the chassis organism could result in a poor target compound yield, side-products accumulation, and/or toxicity to the host [172]. Therefore, current synthetic biology schemes emphasize the proper compartmentalization of heterologous biosynthetic pathways to ensure their proper function in microbial hosts (Fig. 4c).

Localization-aware metabolic engineering in eukaryotic host microbes has enhanced production of isoprenoids [173–177], alkaloids [178, 179], and fatty acid derivatives [180, 181] in various organelles. The value of proper pathway compartmentalization in synthetic biology is probably best exemplified by the recent reconstitution of the tropane alkaloid pathway in baker’s yeast [182]. In this systematic engineering scheme, the authors heterologously expressed >20 carefully selected biosynthetic genes from a number of plant and microbe genomes to rebuild the tropane alkaloid pathway in different cellular compartments. During this process, the authors converted the littorine synthase (LS) into an artificial transmembrane protein to bypass the impediment in maturation and trafficking of the natural plant LS protein through yeast *trans*-Golgi network, a process that is required for the final production of the target compounds. To further facilitate the function of this engineered LS in yeasts, the matured protein was targeted to yeast vacuoles, which better mimics the plant tonoplast environment, where this enzyme naturally functions. The tropine substrates were transported into yeast vacuoles by an additional transporter protein encoded by a tobacco gene. In another recent example, the astaxanthin pathway was targeted to three compartments in *Y. lipolytica* to bring astaxanthin biosynthetic enzymes into close proximity with their precursor compound β-carotene [183]. This compartmentalization-facilitated physical proximity accelerated the conversion of β-carotene into astaxanthin, while significantly decreased the accumulation of metabolic intermediates during this process.

## Concluding remarks and prospectives

From mediating plant–environment interactions to bringing flavor and health to humans, plant SMs play pivotal roles in our ecosphere and societies. Continued genetic dissection of the biosynthetic pathways and optimization of heterologous production systems are required for sustainable exploration and supply of high-value plant SMs to fulfill the ever-growing demand. Current technological advances in genome editing and high-resolution mass spectrometry-based metabolomics have in turn promoted functional studies of plant SMs in ecological contexts. For instance, accelerated biosynthetic gene identification and subsequent production of targeted genetic mutants have facilitated examination of the ecological function of specific plant SMs on a single-pathway or single-compound resolution. On a more systematic scale, integration of mass spectra-based molecular network and information theory statistics can be adapted to test classic ecological theories, as recently been demonstrated with wild tobacco populations [184].

With the powerful tools of current multi-omics analytics and synthetic biology, plant biologists are marching into the uncharted biochemical diversity that remains in the enormous number of non-model species, and developing a variety of chassis organisms for efficient production of different plant SMs. We expect these modern technologies to bring new light into the utilization of plant-specialized metabolism for human health and well-beings.
